# Platelet-Depletion of Whole Blood Reveals That Platelets Potentiate the Release of IL-8 From Leukocytes Into Plasma in a Thrombin-Dependent Manner

**DOI:** 10.3389/fimmu.2022.865386

**Published:** 2022-04-04

**Authors:** Huy Quang Quach, Christina Johnson, Karin Ekholt, Rakibul Islam, Tom Eirik Mollnes, Per H. Nilsson

**Affiliations:** ^1^ Department of Immunology, Oslo University Hospital Rikshospitalet, University of Oslo, Oslo, Norway; ^2^ Research Laboratory, Nordland Hospital, K.G Jebsen Center TREC, University of Tromsø, Bodø, Norway; ^3^ Centre of Molecular Inflammation Research, Department of Cancer Research and Molecular Medicine, Norwegian University of Science and Technology, Trondheim, Norway; ^4^ Department of Chemistry and Biomedicine, Linnaeus Centre for Biomaterials Chemistry, Linnaeus University, Kalmar, Sweden

**Keywords:** platelets, IL-8, acute inflammation, human whole blood, thrombin, cytokines

## Abstract

**Objective:**

In a recent study, we found an elevated level of interleukin 8 (IL-8) in response to bacterial incubation in thrombin-sufficient human whole blood anticoagulated by the fibrin polymerization blocking peptide GPRP. Whether thrombin directly activated leukocytes or mediated the release *via* thrombin-dependent activation of platelets remains unresolved. Herein, we addressed the role of thrombin and platelets in IL-8 release.

**Methods:**

We separated platelets from whole blood using a combination of 0.7% (w/v) citrate and GPRP for attenuating the hemostatic response during the separation of platelets. Cytokine responses were compared in whole blood and platelet-depleted blood upon *Escherichia coli* incubation. Cytokine responses were also profiled with and without reconstitution of either platelets or the supernatant from activated platelets.

**Results:**

Platelets were not activated during the separation process but responded to stimuli upon re-calcification. Plasma levels of IL-1β, IL-1Ra, IL-6, IL-8, IP-10, MIP-1α, and MIP-1β were significantly reduced in platelet-depleted blood compared to whole blood, but recovered in the presence of platelets, or with the supernatant of activated platelets. The leukocyte fraction and platelets were each found to contribute to the elevation of IL-8 at around 5 ng/ml; however, if combined, the release of IL-8 increased to 26 ng/ml. This process was dependent on thrombin since the levels of IL-8 remained at 5 ng/ml in whole blood if thrombin was blocked. Intracellular staining revealed that monocytes were the main source for IL-8 expression.

**Conclusion:**

Our findings suggest that the release of IL-8 is mediated by the leukocytes, mainly monocytes, but potentiated *via* thrombin-dependent activation of platelets.

## Introduction

Platelets are vital for hemostasis and inflammation (1, 2). Conceptually, primary hemostasis is the act of platelet adhesion and aggregation to seal vascular injury by forming a platelet plug (1, 2), while the processes of platelet activation and granule secretion modulate coagulation as well as inflammatory responses (3–6). To study platelets as modulators of inflammation, there is a need to silence platelet functions in whole blood and compare the responses in whole blood with and without platelet contribution. This can be achieved by attenuating platelet activation or separating platelets from whole blood. The advantage of the latter approach is that platelets can be reconstituted back to the same whole blood at later stages after being stored in an inert milieu. However, separating platelets from whole blood needs to be performed delicately without activating the platelets or affecting other blood components to preserve mutual interactions between all blood constituents. Many current methods to purify platelets are suboptimal since they employ inhibitors like citrate in high concentrations or ethylenediamine tetra-acetic acid (EDTA), all of which can alter or even damage platelet functions and influence downstream inflammatory processes (4, 7–9). Citrate is one of the most commonly used anticoagulants (10). While citrate does not interact with proteins *per se*, it does lower the physiological concentration of Ca^2+^ ions (11), inhibiting catalytic activities of proteases requiring Ca^2+^, e.g., the conversion of prothrombin to thrombin by factor (F) Xa in blood coagulation (7). Thrombin is a potent activator of platelets *via* protease-activated receptors (PAR) 1 and 4, a pathway that is inhibited in citrated whole blood (12). Platelet function has also been attenuated in citrate-anticoagulated whole blood in response to activators other than thrombin (13); thus, re-calcification is required to activate isolated platelets for the downstream study of platelet function (10).

The advantage of using a whole blood model for acute inflammation studies is that blood cells and protein cascades can mutually interact in their physiological environments (14). We have extensively investigated innate immunity activation in whole blood anticoagulated with the thrombin inhibitor lepirudin (15), blocking the last step before fibrinogen is activated and keeping all other inflammatory systems, including the complement system, open to mutually interact. Still, however, the limitation of this model is that thrombin is blocked. Recently, we have characterized an *ex vivo* whole blood model using the fibrin polymerization blocking peptide Gly-Pro-Arg-Pro (GPRP) as the anticoagulant (16). Since GPRP exerts its effect downstream of thrombin generation in the coagulation cascade, it allows the study of the crosstalk between coagulation and complement from the node of thrombin (14). The thrombotic response is the primary determinant separating these two *ex vivo* whole blood models; the lepirudin-based model exhibits no active thrombin and low level of platelet activation while thrombin is fully active in the GPRP-based model, allowing platelet activation.

The release of inflammatory cytokines is a cornerstone of the cellular inflammatory responses to pathogenic stimuli (17). Interleukin (IL)-8 represents a vital chemoattractant for leukocyte recruitment (18, 19). It is secreted by various cells, including monocytes, macrophages, and endothelial cells. We and others have previously shown that plasma levels of IL-8 increased in response to complement activation (20). Further on, we recently found that IL-8 release was potentiated in the presence of thrombin (Johnson et al., *submitted manuscript*). However, whether thrombin activates IL-8-producing cells directly or *via* platelet activation remains unclear. Thus, the aim of the present study was to investigate the contribution of platelets in the release of IL-8 to plasma in a whole blood environment by using the advantage of the GPRP-based whole blood model supplemented with a low amount of citrate for gentle separation of platelets.

## Materials and Methods

### Materials

Lepirudin (Refludan^®^) was obtained from Celgene (Uxbridge, United Kingdom). GPRP peptide (Pefabloc^®^ FG or Pefa-6003) was purchased from Pentapharm (Basel, Switzerland). Trisodium citrate was isolated from BD Vacutainer citrate blood sampling tubes (BD, Plymouth, United Kingdom). Heat-inactivated *Escherichia coli* (*E. coli*, strain LE392, catalog no. ATCC 33572) was from American Type Culture Collection (ATCC, Manassas, VA). Thrombin-antithrombin (TAT) ELISA kit (catalog no. OWMG15) was purchased from Siemens Healthcare Diagnostics Products GmbH (Marburg, Germany) while β-thromboglobulin (βTG) ELISA kit (catalog no. 00950) was obtained from Diagnostica Stago, Inc. (Parsippany, NJ).

### Blood Collection

Blood from healthy human donors was obtained by forearm venipuncture using a 21-gauge needle. Informed written consent was obtained from all blood donors. The whole blood (4.5 ml) was collected in polypropylene tubes containing 0.5 ml of either one of the following solutions: i) 0.7% (w/v) citrate, ii) 3.2% (w/v) citrate, iii) the thrombin inhibitor lepirudin (50 μg/ml, final concentration) supplemented with 0.7% (w/v) citrate, or iv) GPRP (8 mg/ml, final concentration) supplemented with 0.7% (w/v) citrate. The volume ratio of blood to anticoagulant was kept at 9:1. An aliquot (100 μl) of whole blood was used for blood cell counting immediately after collection using CELL-DYN Emerald 22 hematology analyzer (Abbott, IL). Experiments with the blood of each donor were performed in triplicates.

### Platelet Isolation and Depletion From Human Whole Blood

The anticoagulated whole blood was centrifugated at 180×*g* for 15 minutes at 25°C without the brake applied. The liquid phase in upper layer, i.e., the platelet-rich plasma (PRP) layer, was gently transferred to 1.5 ml conical polypropylene tubes without disturbing the buffy layer. Tempered (25°C) modified Dulbecco’s phosphate-buffered saline without Ca^2+^ and Mg^2+^ (PBS, Sigma-Aldrich, MO) was added to the residual blood to compensate for the volume of the PRP removed. An aliquot (100 μl) of the residual blood was taken for cell counting. The blood underwent three washing cycles, each by centrifugation at 180×*g* for 15 minutes followed by platelet removal, to further reduce the number of residual platelets. After each step of washing, the blood volume was regained by adding PBS. An aliquot (100 μl) of the remaining blood was taken for cell counting after each step.

The separated PRP was centrifugated at 2000×*g* for 15 minutes at 25°C; the 2/3 top portion of the centrifugated PRP (i.e., platelet-poor plasma) was transferred back to the residual platelet-depleted blood after the last step of washing. The platelet containing portion of the PRP, i.e., the 1/3 bottom, was isolated as the platelet suspension. All cell counts and mean platelet volume (MPV) were measured using CELL-DYN Emerald 22 hematology analyzer (Abbott).

### Conditions for Preparation and Incubations

For all steps in the platelet separation from whole blood, the blood was anticoagulated with 3.2% (w/v) citrate, otherwise with either lepirudin or GPRP supplemented with 0.7% (w/v) citrate. The temperature during the preparation was kept at 25°C. Before platelet and whole blood activation, the 0.7% (w/v) citrate was reversed by the addition of 6.25 mM CaCl_2_ (Sigma-Aldrich) and 3.2% (w/v) citrate was reversed with 25 mM CaCl_2_. The concentration of citrate, which was sufficient to dampen the hemostatic response in GPRP whole blood, was determined by adding citrate of concentrations from 0.2% (w/v) to 3.2% (w/v) to GPRP-whole blood and analysing the expression of CD62P and CD63 on platelets after 15 minutes of incubation. All incubations for functional tests of platelets were carried out at 37°C without or with the addition of *E. coli* (10^7^/ml). After incubation, 16 μl of a stop solution containing a mixture of a CTAD solution [0.08 M trisodium citrate, 11 M theophylline, 2.6 M adenosine, 0.14 M dipyridamole] and 0.14 M EDTA was added to every 100 μl of blood or plasma.

### Detection of Platelet Activation by Flow Cytometry

Platelet activation was quantified by the upregulation of their surface markers, CD63 and CD62P, by flow cytometry. Briefly, after separation from whole blood, the platelet suspension was kept at 25°C for 4 hours, followed by re-calcification and incubation with *E. coli* for 15 minutes at 37°C. Then, platelets were stained for 30 minutes in the dark at 4°C with an antibody mixture containing platelet CD42a-FITC (catalog no. 348083, BD Biosciences, San Jose, CA), CD63-PE-Cy7 (catalog no. 25-0639-42, Invitrogen, Carlsbad, CA), and CD62P-PE (catalog no. 555524, BD Biosciences). After staining, the solution was centrifuged at 250×*g*, 4°C for 5 minutes. The supernatant was discarded and the pellet was resuspended and fixed in PBS containing 0.1% paraformaldehyde (PFA) and 0.1% bovine serum albumin (PBSA) and stored at 4°C until analysis with flow cytometer Attune NxT Acoustic Focusing Cytometer (Thermo Fisher Scientific) within 24 hours from sampling. Platelets were gated as CD42a+ population (**Supplementary Figure 1**) while CD63 and CD62P were used as platelet activation measures. Flow data analysis was performed with FlowJo software version 10 (Ashland, OR).

### Detection of Thrombin and Platelet Activation Markers in Plasma

Thrombin activation was characterized by the quantification of TAT-complexes in plasma, and platelet activation was determined by the level of plasma βTG. Briefly, 200 μl of whole blood or platelet-depleted blood was re-calcified and incubated with or without *E. coli* (10^7^/ml). PBS was added to the control for volume adjustment. The blood was incubated in a rolling incubator at 37°C for 15 minutes before 32 μl of the stop solution was added. Plasma was collected after centrifugation at 3000×*g*, 4°C for 20 minutes, and stored at -80°C for further analyses.

### Scanning Electron Microscopy Image of Platelets

Platelets in the platelet suspension were spun down at 250×*g* at room temperature for 15 minutes. Platelets were allowed to adhere onto Nunc™ Lab-Tek™ II CC2 chamber slide (Thermo Scientific™, catalog no. 154941). The samples then were fixed in 2% glutaraldehyde, dehydrated through an ethanol series and critical point drying (Polaron E3 Critical Point Drier, Polaron Equipment Ltd, United Kingdom). Next, the samples were sputter-coated with a 30 nm-thick layer of platinum in Polaron E5100 sputter coater before being viewed under a scanning electron microscope (SEM) GeminiSEM 300 (Carl Zeiss Microscopy GmbH, Germany).

### Activation of Monocytes and Granulocytes

Platelet-depleted blood (40 μl) was incubated with *E. coli* (10^7^/ml) in a rolling incubator at 37°C for 15 minutes. After adding 6.4 μl of the stop solution, the leukocytes were stained with antibodies: CD45-Pacific Orange (catalog no. MHCD4530, Invitrogen™), CD14-PerCP (catalog no. 340585, BD Biosciences), CD15-V450 (catalog no. 48-0158-42, Invitrogen™), CD11b-APC/Fire 750 (catalog no. 101262, BioLegend, San Diego, CA), and CD35-Alexa Fluor 647 (catalog no. 1981978, Invitrogen™) for 15 minutes at 4°C. After lysing the red blood cells with fixative-free lysis buffer (Invitrogen™, catalog no. HYL250), the solution was centrifuged at 250×*g*, 4°C for 5 minutes. After centrifugation, the supernatant was discarded and the pellet was resuspended in PBSA. The expression of the abovementioned markers was analysed with Attune NxT Acoustic Focusing Cytometer (Thermo Fisher Scientific). Granulocytes were gated as CD15+ population (**Supplementary Figure 2**), while monocytes were gated as CD15- and CD14+ population (**Supplementary Figure 3**). The activation of granulocytes and monocytes was evaluated by the expression levels of CD11b and CD35 on their surfaces. Flow data analysis was performed with FlowJo version 10.

### Intracellular Detection of IL-8

GPRP-anticoagulated whole blood was immediately supplemented with monensin (GolgiStop^TM^, BD Biosciences, San Jose, CA) at 2 µM final concentration and incubated with bacteria for 2 hours. Then, EDTA was added at a 10 mM final concentration. For each test, 25 µl (for monocyte and granulocyte analyses) or 100 µl (for platelet analyses) of whole blood was lysed and fixed utilising the Cytofix/Cytoperm kit containing GolgiStop^TM^ from BD Biosciences (San Jose, CA). Flow cytometry was performed on a Novocyte Flow Cytometer (ACEA Biosciences Inc., San Diego, CA, USA). Single cells were gated utilizing the forward scatter-area (FSC-A) versus the forward scatter-height (FSC-H) dot plot. Granulocytes were identified in a side scatter (SSC)/CD15 dot plot using anti-CD15 BV605 (BD Biosciences). Monocytes were identified in a SSC/CD14 dot plot from the CD15 negative population using anti-CD14 FITC (BD Biosciences). Platelets were analysed in a separate set-up and gated in a SSC/CD42a dot plot using anti-CD42a FITC (BD Biosciences). Here, monocytes were detected using an anti-CD14 V500 antibody (BD Biosciences). Expression of intracellular IL-8 was detected using anti-IL-8 BV421 (clone G265-8, BD Biosciences and expressed as MFI.

### Cytokine Profiling

To study the role of platelets in the inflammatory response, whole blood was collected with GPRP supplemented with 0.7% (w/v) citrate. One part was kept as whole blood, and the other part was processed according to the protocol above, i.e., prepared as platelet suspension as one part and blood containing plasma and all cells except platelets as a second part. Half of the platelet-plasma suspension was re-calcified and incubated for 15 minutes at 37°C to activate the platelets. The activated platelet-plasma suspension was centrifuged for 15 minutes at 3000×*g*, 25°C to remove platelets by hard pellet formation. From the processed blood fractions, the following conditions were prepared: i) whole blood, ii) platelet-depleted blood, iii) platelet-depleted blood with the addition of platelet-plasma suspension, iv) platelet-depleted blood with the addition of plasma after platelet activation, and v) platelet suspension. All components were re-calcified and incubated with *E. coli* (10^7^/ml) in a rolling incubator at 37°C for 4 hours. Plasma was prepared by centrifugation at 3000×*g*, 4°C for 20 minutes and stored at -80°C until analysis with multiplex technology by MAGPIX Luminex (Bio-Rad Laboratories, Hercules, CA) according to the manufacturer’s protocols. A panel of ten inflammatory cytokines and chemokines (IL-1β, IL-1Ra, IL-6, IL-8, IFN-γ, IP-10, MCP-1, MIP-1α, MIP-1β, and TNF) were analysed.

### Statistical Analysis

Statistical comparisons were calculated using GraphPad Prism 9 (GraphPad Software, CA). Tests employed were paired Kruskal–Wallis one-way analysis of variance. A *p*-value <0.05 was considered statistically significant. All data are presented as mean ± standard deviation (SD).

### Ethics Statement

This study was designed and performed according to the ethical guidelines from the declaration of Helsinki. Informed written consent was obtained from the blood donors. The study was approved by the ethical committee of the Norwegian Regional Health Authority, ethical permit REK#S-04114, 2010/934.

## Results

### Platelet Separation From Whole Blood

A low amount of citrate was used to initially stabilize platelet function and its concentration was titrated into GPRP-anticoagulated blood with platelet expression of CD63 and CD62P as the activation read-outs. The expression level of CD63 and CD62P initially decreased in response to the concentration of citrate supplemented (**Supplementary Figure 4**), and 0.7% (w/v) citrate was the optimal concentration in supplementation in GPRP-anticoagulated blood for the expression lowest of CD63 and CD62P (**Supplementary Figure 4**). Meanwhile, there was no difference in the intensities of CD63 and CD62P in lepirudin-anticoagulated blood without and with 0.7% (w/v) citrate supplemented (**Supplementary Figures 5A, B**). To re-calcify, 6.25 mM CaCl_2_ was required to compensate the Ca^2+^ chelated by 0.7% (w/v) citrate, and 25 mM CaCl_2_ for 3.2% (w/v) citrate.

**Figure 1:**
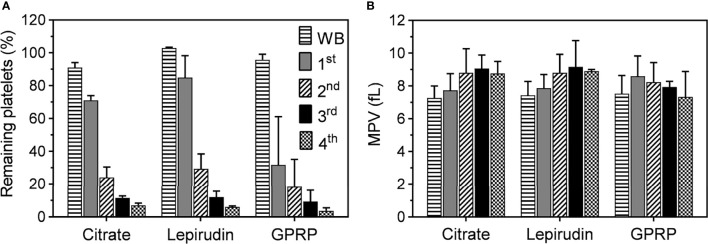
Platelet counts and mean platelet volume (MPV) during the platelet separation process. **(A)** The number of platelets in whole blood immediately after collection (WB) and after the first (1^st^), second (2^nd^), third (3^rd^), and fourth (4^th^) centrifugation. Blood was collected in tubes containing either lepirudin or GPRP, supplemented with 0.7% (w/v) citrate, (labelled in the graphs as “Lepirudin” and “GPRP”) or 3.2% (w/v) citrate only (labelled as “Citrate”), with blood to anticoagulant ratio 9:1. Data are presented as the normalized mean percentage of platelet number ± standard deviation (SD) for each donor (n = 9). **(B)** Mean platelet volume (MPV), as an indicator of platelet integrity, remained at <10 fl throughout the whole purification process. Data are presented as mean of MPV ± standard deviation (SD). Blood from each donor (n = 9) was analysed in triplicates.

Platelet counts and mean platelet volumes (MPV) in blood anticoagulated with 0.7% (w/v) citrate were similar to those in blood anticoagulated with 3.2% citrate (w/v) and either lepirudin or GPRP supplemented with 0.7% (w/v) citrate (**Supplementary Figures 5C, D**). However, blood anticoagulated with only 0.7% citrate clotted within 10 minutes after blood sampling; hence, data from this blood sample condition could not be further collected. Platelets in 0.7% (w/v) citrated blood transformed their morphologies into early dendritic shapes (**Supplementary Figures 5E, F**), signalling early stage of platelet activation (21). No blood clot was formed with 3.2% (w/v) citrate, a condition used in routine haematological laboratories worldwide and included as control in this study. Neither was any clot formed in 0.7% (w/v) citrate supplemented in either lepirudin or GPRP blood sampling conditions. We found that after one centrifugation, 71.1 ± 2.7% of platelets remained in 3.2% citrated blood after removing the PRP layer (**Figure 1A**). These numbers in lepirudin- and GPRP-anticoagulated blood were 84.9 ± 13.3% and 31.7 ± 29.4%, respectively (**Figure 1A**).

Three more washing steps were applied by repeated centrifugation and the number of platelets remaining in the residual blood reduced after each step (**Figure 1A**). At the end (4^th^ centrifugation) of the washing process, 6.1 ± 0.6% and 3.7 ± 1.8% of platelets remained in lepirudin- and GPRP-anticoagulated blood, respectively, while this number in 3.2% citrated blood was 7.0 ± 1.3%. Throughout the process, MPV remained at <10 fl throughout the successive washing steps (**Figure 1B**). In addition, the cell counts of other blood cells, i.e., white and red blood cells, remained mainly unchanged (**Supplementary Figure 6**).

### Activation of Platelets

**Figure 2:**
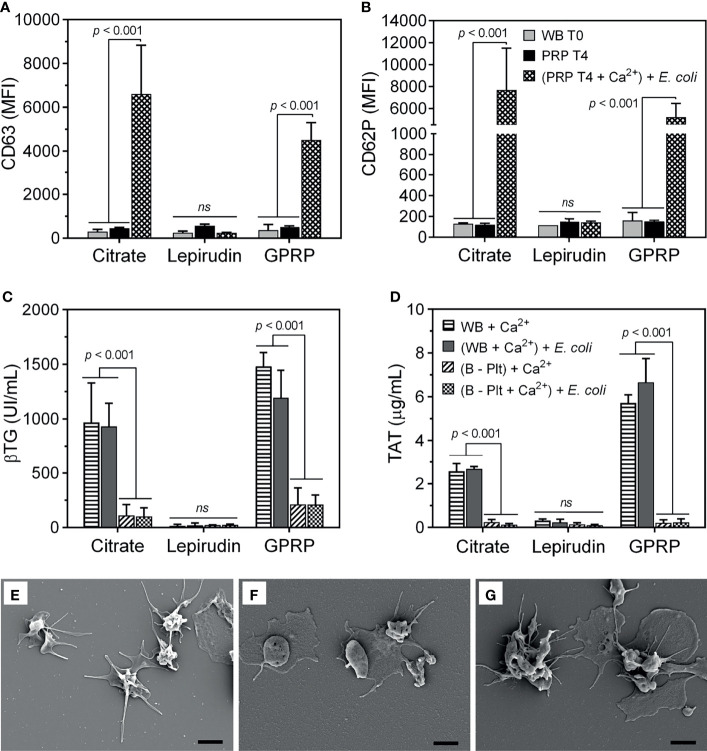
Evaluation of platelet activation during the platelet separation process. The activation of platelets was evaluated by the expression of **(A)** CD63 and **(B)** CD62P on the platelet surface using flow cytometry. The expression of CD63 and CD62P was measured in whole blood immediately after collection (indicated as “WB T0”) and in platelet-rich plasma (PRP) 4 hours after platelet isolation (indicated as “PRP T4”). In addition, 4 hours after platelet separation, platelet activation was induced by incubation with *E*. *coli* (10^7^/ml, indicated as “(PRP T4 + Ca^2+^) + *E*. *coli”* after re-calcification. Data are presented as mean of median fluorescence intensity (MFI) ± standard deviation (SD) (n = 9). **(A, B)** have the same annotation as shown in **(B)**. In addition, platelet activation was evaluated by its soluble activation marker **(C)** β-thromboglobulin (βTG) and thrombin generation by **(D)** thrombin-antithrombin complex (TAT) in plasma from whole blood after blood sampling (WB + Ca^2+^) and whole blood after platelet-depletion [(B - Plt) + Ca^2+^], or after incubation with *E*. *coli* for 15 minutes, indicated as “(WB + Ca^2+^) + *E*. *coli*” and “(B - Plt + Ca^2+^) + *E. coli*”, respectively. Data are represented as mean ± standard deviation (SD) (n = 6). **(C, D)** have the same annotation as shown in **(D)**. Platelet activation was further evaluated with scanning electron microscopy, as presented in an image representative of 6-9 samples collected **(E–G)**. Platelets were collected in plasma-rich plasma (PRP) from whole blood anticoagulated with 3.2% (w/v) citrate **(E)**, lepirudin (50 μg/ml) supplemented with 0.7% (w/v) citrate **(F)**, and GPRP (8 mg/ml) supplemented with 0.7% (w/v) citrate **(G)**. Thrombin was used as a platelet activator. The scale bar at the lower right represents 2 μm. ns, non significant.

In addition to measuring MPV of platelets as an indicator of platelet activation (**Figure 1B**), we further characterized the expression of platelet activation markers on the platelet surface. Both CD63 and CD62P markers from dense and alpha granules, respectively, were detected at background levels immediately after blood collection and remained almost unchanged after preparing the PRP suspension until 4 hours after blood sampling (**Figures 2A, B**), implying minimal activation of the platelets (22). Equally important, platelets retained their functions in response to *E. coli*. Upon re-calcification, incubation with *E. coli* for 15 minutes at 37°C induced a substantial expression of both CD63 and CD62P on platelets in PRP suspension from both citrate 3.2% (w/v) and GPRP (**Figures 2A, B**). In contrast, platelets prepared from lepirudin-anticoagulated blood only expressed background levels of CD63 and CD62P when incubated with *E. coli* (**Figures 2A, B**).

Upon activation, platelets undergo morphological changes and enhance the expression of surface markers like CD63 and CD62P and release alpha granule-stored βTG (23). In 3.2% (w/v) citrate and in GPRP-anticoagulated blood, βTG was detected at ~1000 UI/ml after re-calcification and incubation at 37°C for 15 minutes (**Figure 2C**). After platelet depletion, the level of βTG in 3.2% (w/v) citrate and GPRP-anticoagulated blood dropped to 10 - 15% of the corresponding platelet-intact whole blood (**Figure 2C**). A virtually identical pattern was observed with TAT, which was detected at levels up to 6 µg/ml before platelet removal and decreased to 0.2 µg/ml after platelet depletion (**Figure 2D**). In contrast, βTG and TAT were detected at background levels in lepirudin-anticoagulated blood both before and after platelet depletion (**Figures 2C, D**). The activation of platelets in blood was confirmed under SEM (**Figures 2E–G**). Platelets isolated from 3.2% (w/v) citrate-, lepirudin-, and GPRP-anticoagulated blood were activated under thrombin stimulation, as evidenced by their wide-spreading (**Figures 2E–G**), indicating that platelets retained their functions during the separation process (21).

### Activation of Granulocytes and Monocytes

**Figure 3:**
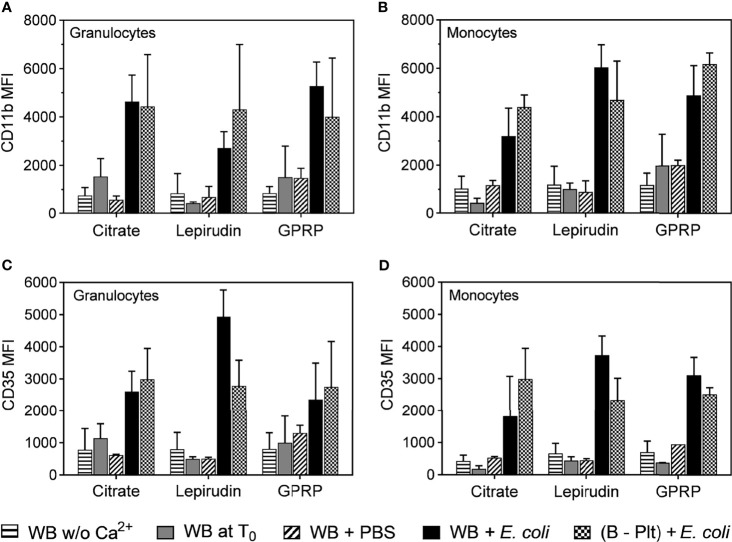
Activation of granulocytes and monocytes during the platelet depletion process. The activation of granulocytes **(A, C)** and monocytes **(B, D)** were evaluated by the surface expression levels of CD11b **(A, B)** and CD35 **(C, D)** by flow cytometry. The activation of these cells was measured in whole blood immediately after collection without re-calcification (indicated as “WB w/o Ca^2+^”) or whole blood with Ca^2+^ added at T0 (WB at T0). In addition, the activation of granulocytes and monocytes in Ca^2+^ supplemented whole blood was incubated with PBS (WB + PBS) or *E. coli* (WB + *E. coli*). After platelet removal, these cells were activated with *E. coli* ((B – Plt) + *E. coli*). All incubations were carried out for 15 minutes after the addition of Ca^2+^. Data are presented as mean of median fluorescence intensity (MFI) ± standard deviation (SD) (n = 9).

Granulocytes and monocytes play an important role in the acute inflammatory response (24, 25). We characterized the activation of these cells in blood before and after platelet separation. The expression level of the activation marker CD11b on both granulocytes and monocytes in all anticoagulants was low immediately after blood sampling and remained unchanged after the blood underwent successive washing steps (**Figures 3A, B**). To confirm that the protocol did not interfere with cell functions, the activation of these cells was evaluated in platelet-depleted blood. *E. coli* at 10^7^/ml induced a 5-fold increase in CD11b expression, which was comparable to the activation levels in whole blood incubated with *E. coli* after collection without centrifugation (**Figures 3A, B**).

Complement receptor 1 or CD35, with C3b as the main ligand, is expressed on the surface of both monocytes and granulocytes, and an up-regulation of CD35 is indicative of the activation status of these cells (26). Similar to CD11b, we found that the expression of CD35 was not affected by the platelet separation protocol and responded to *E. coli* incubation (**Figures 3C, D**).

### Plasma Cytokine Release

**Figure 4:**
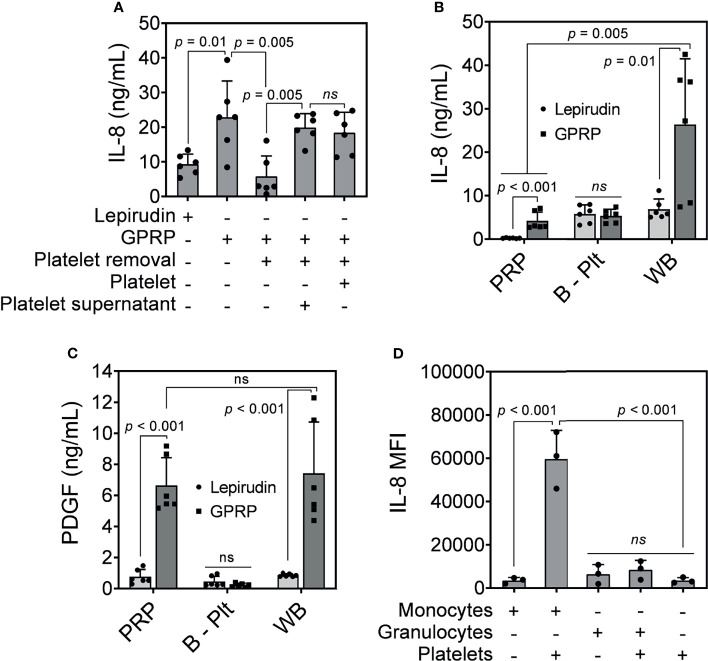
IL-8 release in response to *E. coli*. **(A)** IL-8 was measured in GPRP-anticoagulated blood after incubation with *E. coli* (10^7^/ml) for 4 hours after the addition of Ca^2+^. The IL-8 levels were compared in whole blood, in platelet-depleted blood (“Platelet removal”), in platelet-plasma reconstituted (“Platelet”), and in reconstituted plasma supernatant from activated platelets (“Platelet supernatant”). **(B)** IL-8 and **(C)** PDGF measured in activated platelet-rich plasma (PRP), in platelet-depleted blood (B - Plt) and in whole blood (WB), all anticoagulated with either GPRP or lepirudin. Data are presented as mean ± standard deviation (SD) (n = 6). **(D)** Detection of intracellular IL-8 by flow cytometry in GPRP-anticoagulated blood. Data are presented as mean of median fluorescence intensity (MFI) ± standard deviation (SD) from 3 blood donors with each dot representing average MFI of two replicates from each donor. ns, non significant.

Having shown that our protocol effectively removed platelets from whole blood (**Figure 1**), while not activating platelets (**Figure 2**), granulocytes and monocytes (**Figure 3**), we further profiled the release of ten inflammatory cytokines and chemokines in platelet-depleted blood in response to *E. coli*, including IL-1β, IL-1Ra, IL-6, IL-8, IFN-γ, IP-10, MCP-1, MIP-1α, MIP-1β, and TNF. This panel was selected basing on its elevation in response to *E. coli* in whole blood after a 4-hour incubation. Remarkable reductions were observed in the release of seven cytokines in platelet-depleted blood as compared to whole blood, including IL-8 (**Figure 4A**), IL-1β, IL-1Ra, IL-6, IP-10, MIP-1α, and MIP-1β (**Supplementary Figures 7A–F**). Meanwhile, there was no apparent difference in the release of IFN-γ, MCP-1, and TNF before and after platelet removal (**Supplementary Figures 7G–J**).

IL-8 levels were elevated in GPRP-whole blood in response to *E. coli* and decreased to background level in platelet-depleted blood (**Figure 4A**). However, IL-8 levels in GPRP-anticoagulated blood were resurgent in platelet-depleted blood upon the addition of either the platelet-plasma suspension or only the supernatant from activated platelets (**Figure 4A**). This result indicated that the increased IL-8, which we previously characterized to be dependent on thrombin, was mediated *via* the activation of platelets.

We investigated the role of platelets in the upregulation of IL-8 by measuring plasma levels of IL-8 in PRP, in platelet-depleted blood, and in GPRP-whole blood upon *E. coli* incubation. Interestingly, plasma IL-8 was detected at 4.19 ng/ml in PRP prepared from GPRP anticoagulated blood (**Figure 4B**). Lepirudin was also included here as control and the IL-8-level was detected at 0.22 ng/ml in PRP from lepirudin-anticoagulated blood. Plasma IL-8 was also detected at 5.81 and 5.36 ng/ml in platelet-depleted blood anticoagulated with lepirudin and GPRP, respectively (**Figure 4B**). Notably, IL-8 level increased up to 26.36 ng/ml in whole blood anticoagulated with GPRP (**Figure 4B**).

The activation of platelets was confirmed by a significant increase of platelet-derived growth factor (PDGF) in PRP and whole blood prepared with GPRP (**Figure 4C**) (27). The levels of PDGF were not significantly different between PRP and whole blood prepared with GPRP (**Figure 4C**). In contrast, in platelet-removed blood, PDGF was detected at background levels and there was no difference in PDGF levels between lepirudin- and GPRP-blood (**Figure 4C**).

We further investigated which leukocyte population was the main source of IL-8. Intracellular cytokine staining showed that IL-8 was mainly generated by monocytes (**Figure 4D**). As such, monocytes alone attributed to an average IL-8 intensity of 3485 MFI, which was comparable to that of platelets (3502 MFI) (**Figure 4D**). However, the intensity of IL-8 increased over 17 folds, to 59690 MFI when monocytes were found in conjugation with platelets (**Figure 4D**). Meanwhile, IL-8 in granulocytes was detected at 6417 MFI and increased 1.3 folds to an intensity of 8397 MFI together with platelets (**Figure 4D**).

## Discussion

We have recently characterized the four-amino acid peptide GPRP for the anticoagulation of human whole blood for up to 8 hours at 37°C (16). In this whole blood model, we found that βTG and TAT were instantly generated in the anticoagulated blood during the process of blood sampling. This was dependent on instant activations of the coagulation system and platelets in the blood from contact with the surfaces of the needle, the sampling tube and the contact with air. Thus, in this study, we exploited citrate at low concentration (0.7%, w/v) supplemented in GPRP for controlling the instant thrombin activation. With citrate, we could gently separate platelets from whole blood and study the contribution of platelets in the inflammatory response to *E. coli*. We chose to combine citrate with GPRP since the citrate-dependent chelation of Ca^2+^ ions is reversible (10), and with GPRP, we blocked the coagulation cascade without interfering with thrombin. Altogether, this approach offered a holistic system to study thromboinflammation in an *ex vivo* blood model.

The addition of citrate to GPRP-whole blood prevented the instant platelet activation in a manner dependent on citrate concentration and 0.7% citrate (w/v) was selected for further experiments (**Supplementary Figure 4**). The parallel result was observed in lepirudin-anticoagulated blood supplemented with 0.7% citrate (w/v) (**Supplementary Figure 5**), reflecting the nature of this *ex vivo* blood model. As such, thrombin, as a potent platelet activator and a key component in the coagulation cascade, is blocked in lepirudin-anticoagulated blood (15), and consequently, the addition of citrate did not affect platelet activation. Although 0.7% (w/v) citrate was sufficient to stabilize platelets initially in GPRP-whole blood, it did not completely prevent blood from clotting by itself.

The high percentage of platelets remaining in whole blood after the first centrifugation indicated that one round of 15 minutes centrifugation at 180×*g* was not sufficient to remove all platelets from whole blood. The remaining platelets may exert their effects, leading to an inaccurate conclusion unnoticedly and necessitating a method to completely remove platelets from whole blood. After three successive washing steps, platelets were successfully removed with only <4% of platelets remaining in GPRP-blood supplemented with 0.7% citrate (w/v) (**Figure 1A**). As an indicator of platelet activation, MPV remained at <10 fl (**Figure 1B**), which was within the acceptable range of 7.2 to 11.7 fl (28), implying that platelet integrity was kept intact throughout the washing process (22).

A major concern during the separation of platelets is that platelet activation may be induced unintentionally. In addition to acceptable MPV (**Figure 1B**), background levels of both CD63 and CD62P suggested low activation levels of platelets, if any, from blood sampling up to 4 hours of storing platelets in PRP (**Figures 2A, B**). Upon incubation with *E. coli*, separated platelets from 3.2% (w/v) citrate and GPRP supplemented with 0.7% (w/v) citrate blood activated normally as evidenced in significant increases of CD63 and CD62P (**Figures 2A, B**) and their well-spread morphologies in SEM images (**Figures 2E–G**) (29). These responses suggested that platelets retained their functions during the separation process. Background levels of βTG and TAT detected in blood after platelet removal when incubated with *E. coli* confirmed a minimal residual number of platelets (**Figures 2C, D**), which was consistent with the low platelet counts in platelet-depleted blood observed earlier (**Figure 1A**). Platelets prepared from thrombin inhibitor lepirudin-anticoagulated blood only expressed background levels of CD63 and CD62P even in incubation with *E. coli* (**Figures 2A, B**), confirming the role of thrombin as a crucial platelet activator (12). Taken together, these results suggested that the whole separation process did not induce detectable activation of the platelets, and the separated platelets retained their functions by responding to stimuli.

Our method of platelet removal from whole blood was based on repeated centrifugations, which may induce activation of other circulating cells in the blood, especially granulocytes and monocytes, which are critically involved in the acute inflammatory response (24, 25). CD11b/CD18 (complement receptor 3, CR3) is involved in cellular adhesion and migration, and is the most important complement phagocytosis receptor using iC3b as a ligand (30). CD11b is rapidly increased during leukocyte activation; hence, a low expression of CD11b observed in both granulocytes and monocytes correlated with a low level of activation of these cells (**Figures 3A, B**). Similarly, low expression of CD35 or complement receptor 1, with C3b as its main ligand, suggested a low activation status of granulocytes and monocytes (**Figures 3C, D**) (26). However, the expression of both CD11b and CD35 in platelet-removed blood increased up to the levels of those in whole blood in response to *E. coli* incubation, confirming these cells were functional after the platelet removal process (**Figure 3**).

Among the seven cytokines that were platelet-dependent (**Figure 4A** and **Supplementary Figures 7A–F**), IL-1β is crucially dependent on inflammasome activation; IL-6 are typical of early pro-inflammatory mediators; IL-10 and IL-1Ra are typically anti-inflammatory; whereas IL-8, MIP-1α, and MIP-1β are highly potent chemokines. Interestingly, the levels of these cytokines were resurgent by adding back to platelet-removed blood either separated platelets or the supernatant of activated platelets. This resurgence implied that the activation of platelets serves as a trigger for the release of these cytokines in whole blood upon *E. coli* incubation.

Also shown as platelet-dependent cytokine (**Figure 4A**), IL-8 was detected at 4.1 ± 1.9 ng/ml in the PRP portion of GPRP-anticoagulated blood and at 0.2 ± 0.1 ng/ml in the PRP portion of lepirudin-anticoagulated blood (**Figure 4B**), suggesting that platelets released IL-8 in a thrombin-dependent manner. Higher than background levels of IL-8 detected in platelet-removed blood indicated that IL-8 was also released or stored by other cells and this IL-8 source was not dependent on platelets (**Figure 4B**). However, IL-8 detected in GPRP-whole blood was significantly higher than those in the PRP portion of GPRP-blood and lepirudin-whole blood, meaning that platelets potentiated the release of IL-8 from leukocytes. Both platelets and leukocytes can independently release IL-8 into plasma, but for the leukocytes to produce and release IL-8 at higher levels, it needs the potentiation from platelets. The activation of platelets was confirmed with a significant increase of PDGF in both the PRP portion and whole blood anticoagulated with GPRP compared to lepirudin (**Figure 4C**). The elevation of IL-8 in monocytes, but not granulocytes, when incubated with platelets further revealed monocytes as a main source of IL-8. Combined with the resurgence of IL-8 in platelet-removed blood with the addition of either platelets or the supernatant from activated platelets (**Figure 4A**) and intracellularly detected IL-8 (**Figure 4D**), these results supported monocytic origin of the IL-8 release; however, this increase was triggered in response to activated platelets.

In conclusion, we present here an approach to separate and, in parallel, deplete platelets from human whole blood using GPRP in combination with low levels of citrate as an anticoagulant. This method removed 96% of the platelets in whole blood, mitigating their effects in the inflammatory response in whole blood. This method showed remarkably low background activity during the process, and with platelets not undergoing methodological activation. We unravelled the essential role of platelets in potentiating bacterial-induced IL-8 release from monocytes in human whole blood.

## Data Availability Statement

The original contributions presented in the study are included in the article/**Supplementary Material**. Further inquiries can be directed to the corresponding author.

## Ethics Statement

This study was designed and performed according to the ethical guidelines from the declaration of Helsinki. Informed written consent was obtained from the blood donors. The study was approved by the ethical committee of the Norwegian Regional Health Authority, ethical permit REK#S-04114, 2010/934. The patients/participants provided their written informed consent to participate in this study.

## Author Contributions

HQ conceived of the presented idea. HQ, CJ, KE, and RI carried out the experiments. HQ wrote the manuscript with support from CJ, TM, and PN. TM and PN supervised the project. All authors contributed to the article and approved the submitted version.

## Funding

This study was financially supported by The Norwegian Research Council (Project No. 274332), The Swedish Research Council (Project No. 2018-04087), The Norwegian Council on Cardiovascular Disease, The Odd Fellow Foundation, The Simon Fougner Hartmann Family Fund, and The Crafoord Foundation (20190890 and 20210961).

## Conflict of Interest

The authors declare that the research was conducted in the absence of any commercial or financial relationships that could be construed as a potential conflict of interest.

## Publisher’s Note

All claims expressed in this article are solely those of the authors and do not necessarily represent those of their affiliated organizations, or those of the publisher, the editors and the reviewers. Any product that may be evaluated in this article, or claim that may be made by its manufacturer, is not guaranteed or endorsed by the publisher.
