# Supplementary material for: Platelet-Depletion of Whole Blood Reveals That Platelets Potentiate the Release of IL-8 From Leukocytes Into Plasma in a Thrombin-Dependent Manner
Source: Front Immunol. 2022 Apr 4;13:865386. doi: 10.3389/fimmu.2022.865386 (PMC9013889; doi:10.3389/fimmu.2022.865386)
Supplement: Supplementary file 1 [file DataSheet_1.docx]

**Supplementary Figures**

**Platelet-depletion of whole blood reveals that platelets potentiate the release of IL-8 from leukocytes into plasma in a thrombin-dependent manner**

Huy Quang Quach^1^, Christina Johnson^1^, Karin Ekholt^1^, Rakibul Islam^1^, Tom Eirik Mollnes^1,2,3^, Per H. Nilsson^1,4^

^1^Department of Immunology, University of Oslo and Oslo University Hospital Rikshospitalet, Sognsvannsveien 20, 0372 Oslo, Norway

^2^Research Laboratory, Nordland Hospital, and K.G Jebsen Center TREC, University of Tromsø, Prinsens Gate 164, 8092, Bodø, Norway

^3^Centre of Molecular Inflammation Research, and Department of Cancer Research and Molecular Medicine, Norwegian University of Science and Technology, Trondheim, Norway ^4^Department of Chemistry and Biomedicine, Linnaeus Centre for Biomaterials Chemistry, Linnaeus University, Kalmar, Sweden

**
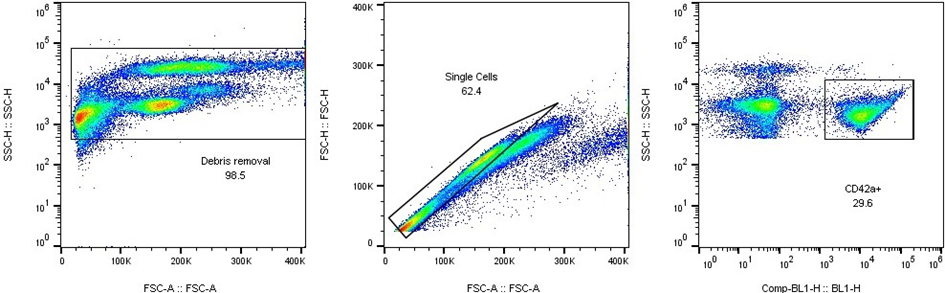
**

**Supplementary Figure 1. Gating strategy for platelets.** Platelets were initially gated as single cells, excluding doublets on a dot-plot of forward scatter area (FSC-A) versus forward scatter height (FSC-H) and subsequently gated CD42a+ (BL-1) population.


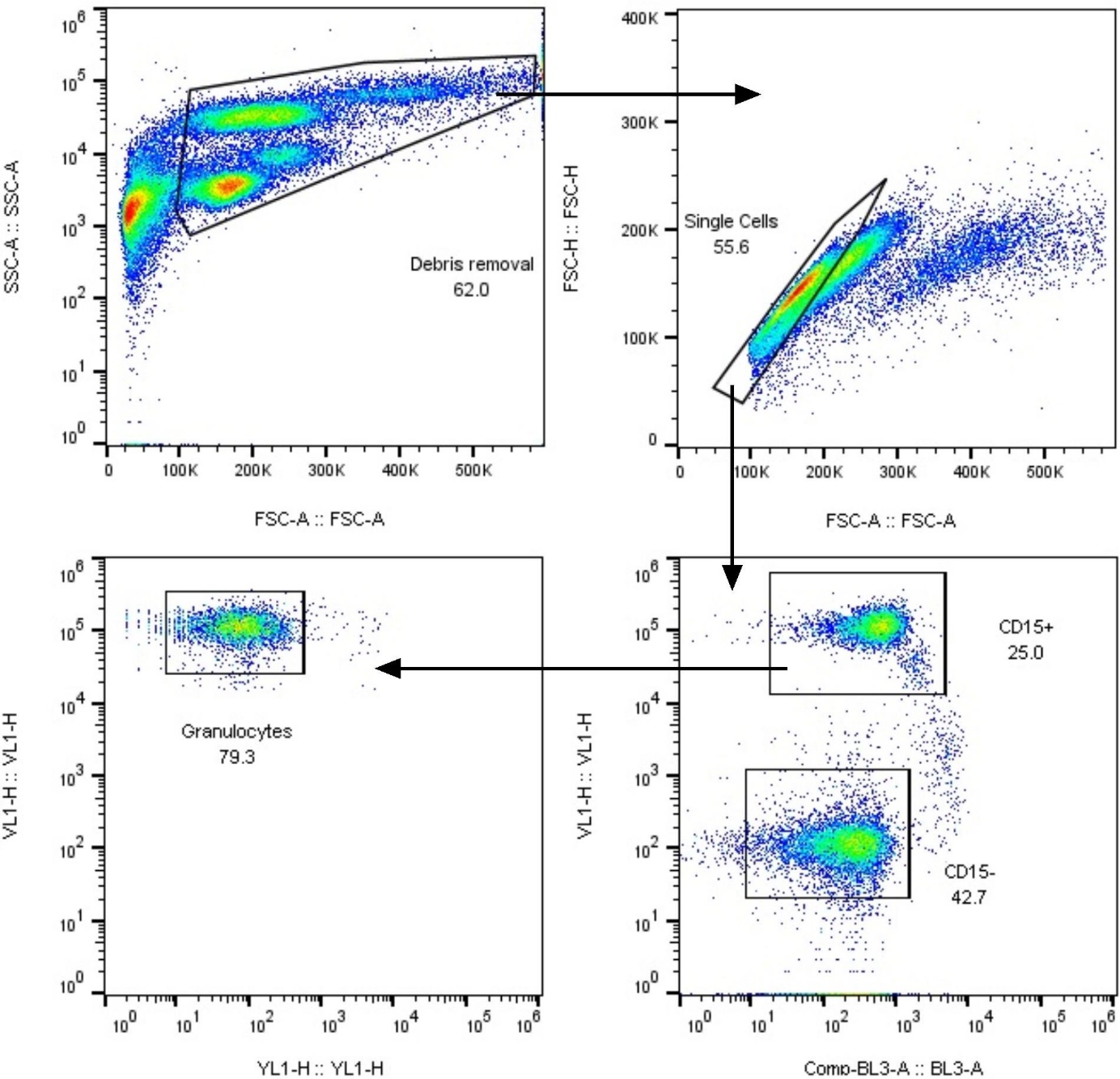


**Supplementary Figure 2. Gating strategy for granulocytes.** Granulocytes were initially gated as single cells, excluding doublets on a dot-plot of forward scatter area (FSC-A) versus forward scatter height (FSC-H) and subsequently gated CD15+ (VL-1) and CD14- (BL-3) population. Granulocytes were further confirmed as CD62P- (YL-1) population.


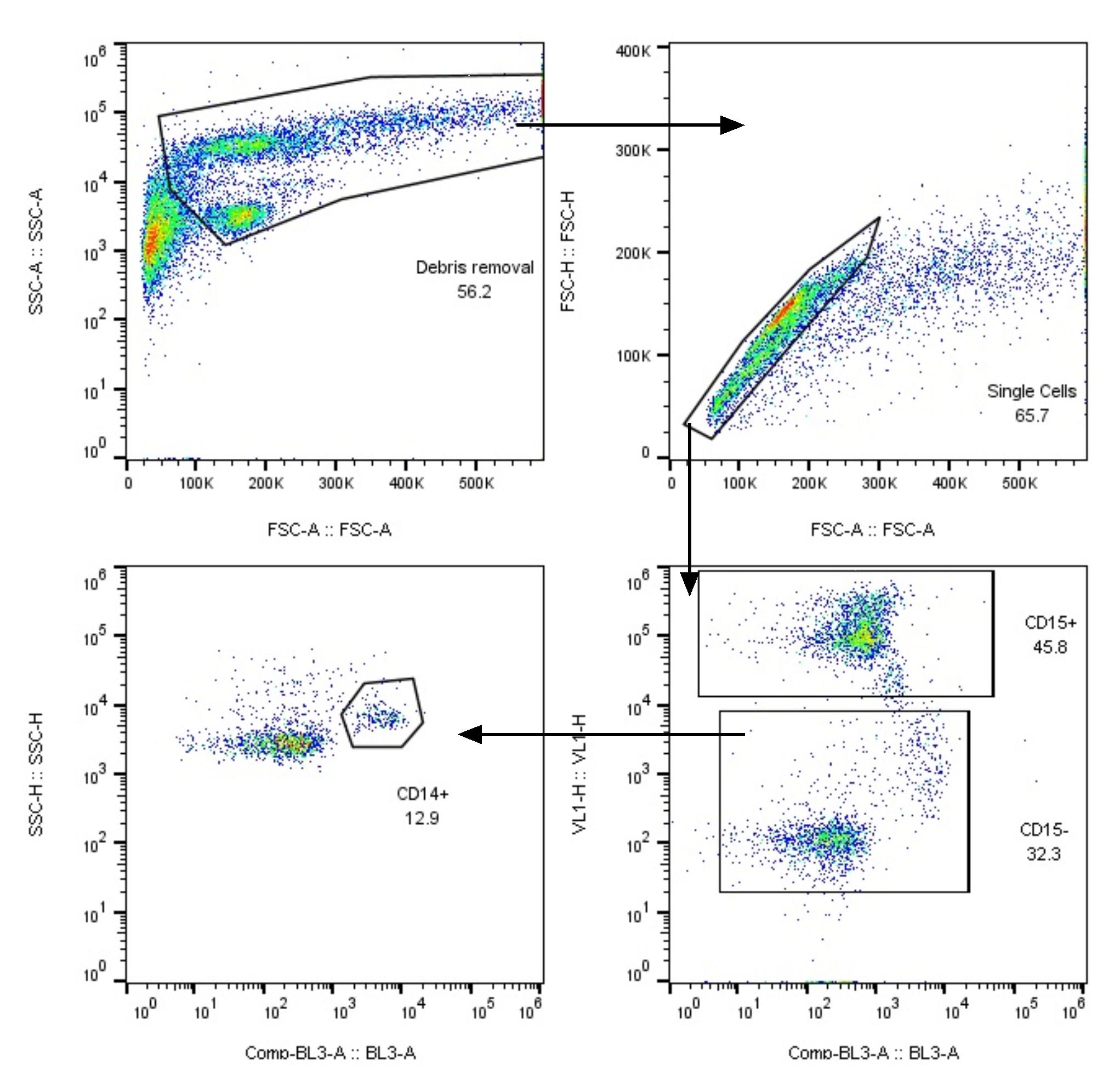


**Supplementary Figure 3. Gating strategy for monocytes.** Monocytes were initially gated as single cells, excluding doublets on a dot-plot of forward scatter area (FSC-A) versus forward scatter height (FSC-H) and subsequently gated as CD15- (VL-1) and CD14+ (BL-3) population.


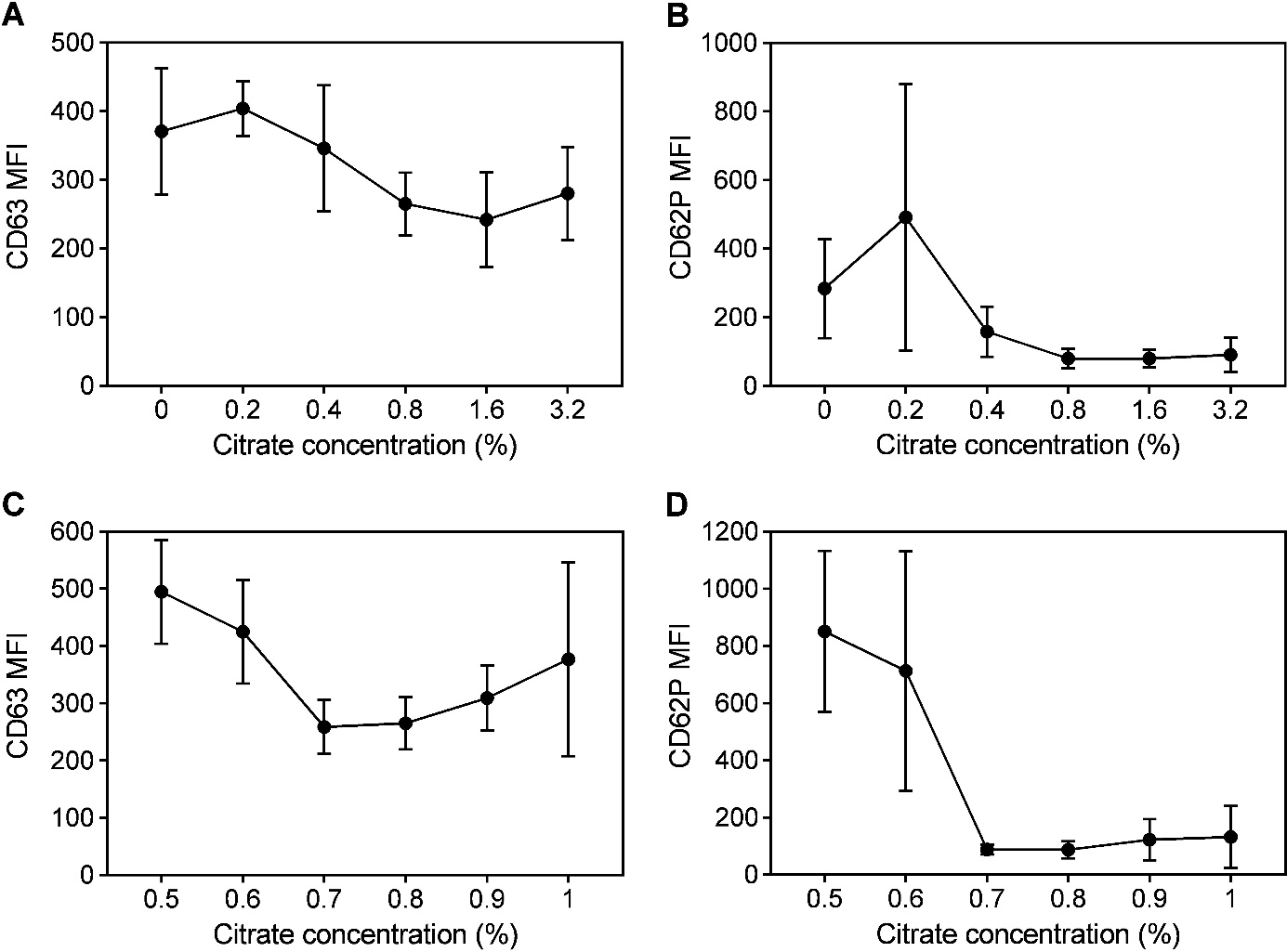


**Supplementary Figure 4. Citrate-dependent platelet activation in GPRP anticoagulated whole blood.**  The concentration of citrate was varied from 0 to 3.2% (A and B), and further closely titrated (C and D) in the range of 0.5 to 1.0% (C and D). Platelet activation was quantified by surface expression levels of CD63 and CD62P by flow cytometry. Data are presented as median fluorescence intensity (MFI) ± standard deviation (SD) (n = 6).


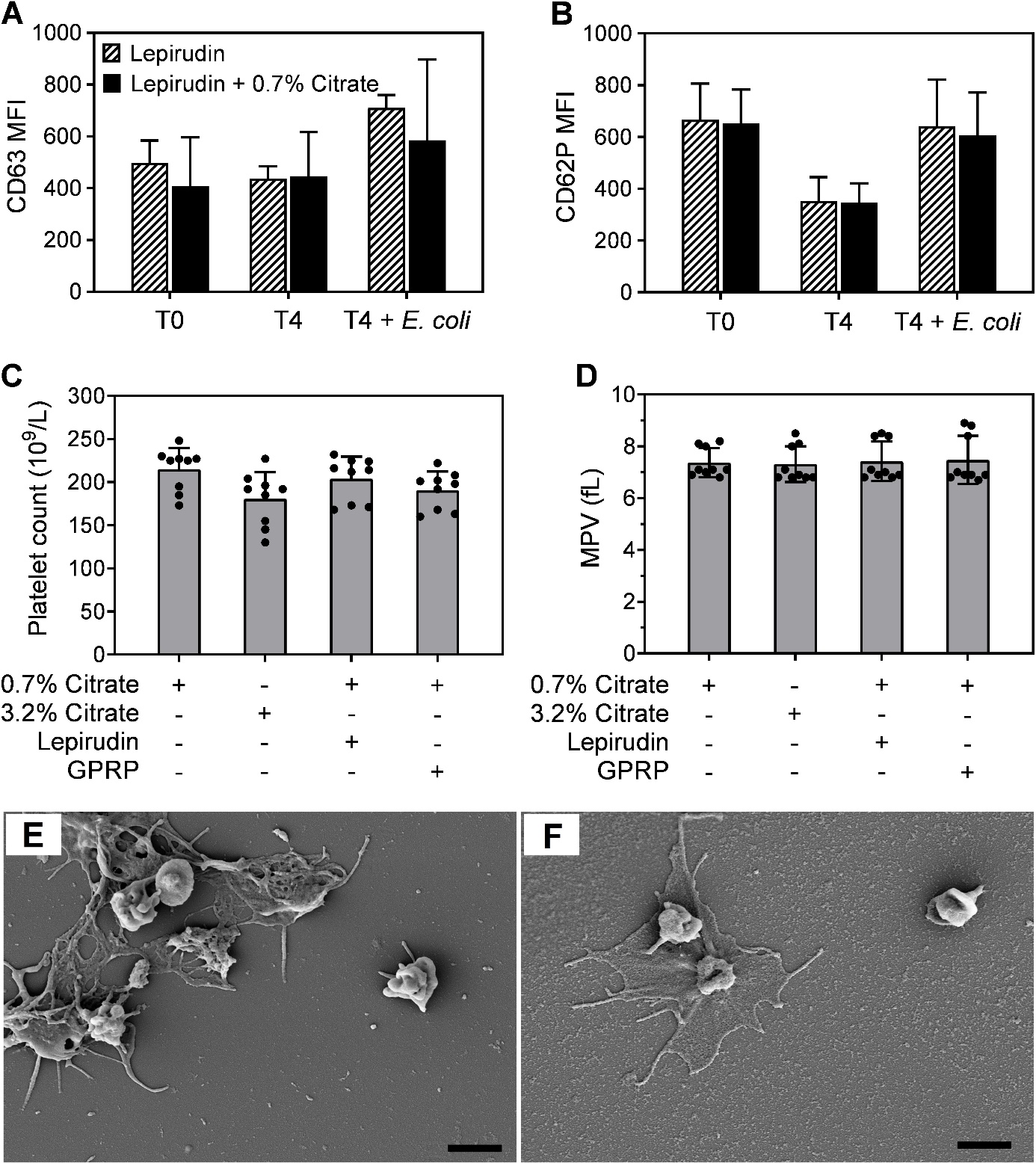


**Supplementary Figure 5. Platelet activation and platelet count.** (A) and (B) Platelet activation in whole blood anticoagulated with lepirudin and lepirudin supplemented with 0.7% citrate. Platelet activation was quantified by surface expression levels of CD63 (A) and CD62P (B) by flow cytometry. Data are presented as mean of median fluorescence intensity (MFI) ± standard deviation (SD) (n = 6). (C) Platelet counts and (D) mean platelet volume (MPV) from whole blood immediately after collection. Blood was anticoagulated under either one of these conditions: i) 0.7% citrate, ii) lepirudin (50 μg/ml, final concentration) supplemented with 0.7% citrate, iii) GPRP (8 mg/ml, final concentration) supplemented with 0.7% citrate, or iv) 3.2% citrate. (E) and (F) Scanning electron microscopy (SEM) of platelets. SEM images of platelets separated from whole blood anticoagulated with 0.7% citrate. SEM images were taken from platelets separated immediately after collection of blood (E), or after separation and being activated with thrombin (F). The scale bar at the lower right represents 2 μm.


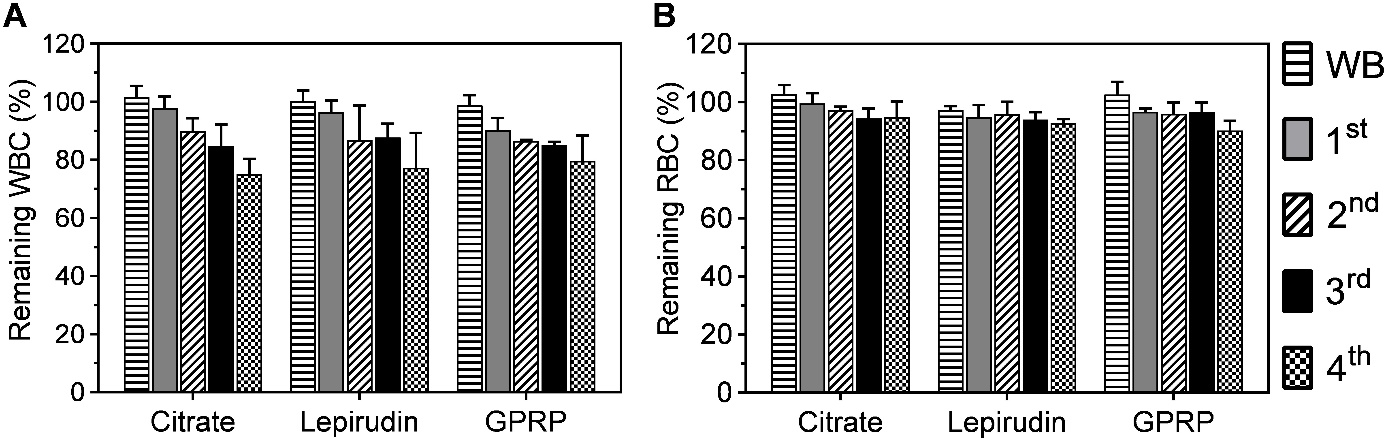


**Supplementary Figure 6**. **White blood cell (WBC) and red blood cell (RBC) counts during the platelet depleting process**. (A) WBC and (B) RBC remained in whole blood counted immediately after collection (WB) and after the first (1^st^), second (2^nd^), third (3^rd^), and fourth (4^th^) centrifugation. Data are presented as the normalized mean of cell count ± standard deviation (SD) (n = 9).


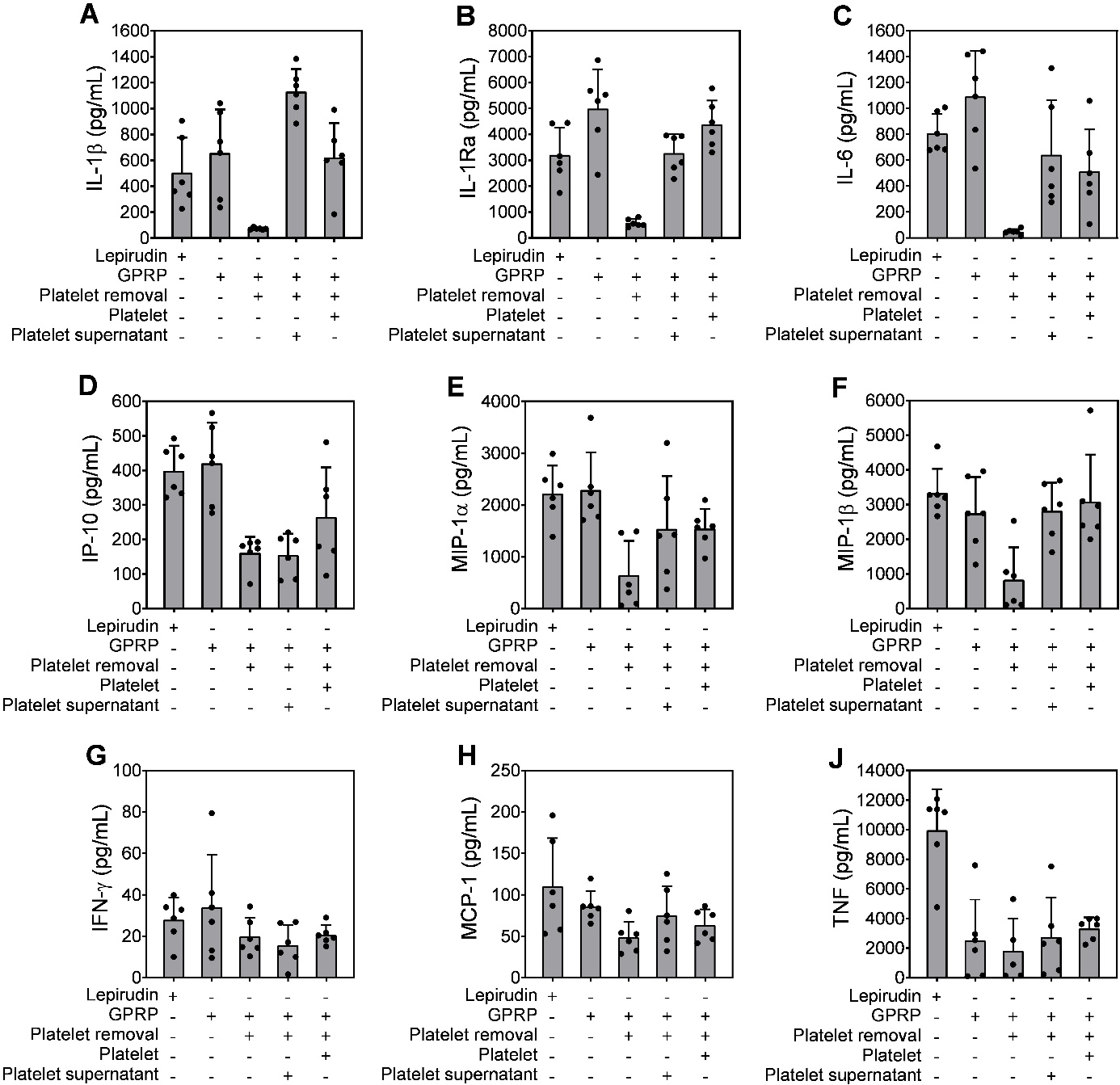


**Supplementary Figure 7**. **Plasma cytokine release induced by *E. coli***. A panel of cytokines was measured in GPRP-anticoagulated blood after incubation with *E. coli* (10^7^/ml) for 4 hours at 37^o^C. The levels in whole blood, platelet-depleted whole blood (“Platelet removal”), platelet-plasma reconstituted (“Platelets”) and reconstituted plasma supernatant from activated platelets (“Platelet supernatant”) were compared. Data are presented as mean ± standard deviation (SD) (n = 6).
